# Nationwide changes in physical activity, nutrient intake, and obesity in South Korea during the COVID-19 pandemic era

**DOI:** 10.3389/fendo.2022.965842

**Published:** 2022-09-13

**Authors:** Hong Jun Yang, Saengryeol Park, Tai-Young Yoon, Jae-Hong Ryoo, Sung Keun Park, Ju Young Jung, Ju-Hyung Lee, Chang-Mo Oh

**Affiliations:** ^1^ Institute of Health and Environment, Graduate School of Public Health, Seoul National University, Seoul, South Korea; ^2^ Department of Physical Education, School of Education, Chonnam National University, Gwangju, South Korea; ^3^ Department of Preventive Medicine, School of Medicine, Kyung Hee University, Seoul, South Korea; ^4^ Department of Occupational and Environment Medicine, School of Medicine, Kyung Hee University, Seoul, South Korea; ^5^ Total Healthcare Center, Kangbuk Samsung Hospital, Sungkyunkwan University, School of Medicine, Seoul, South Korea; ^6^ Department of Preventive Medicine, School of Medicine, Jeonbuk National University, Jeonju, South Korea

**Keywords:** COVID-19, obesity, exercise, physical activity, nutrients, diet

## Abstract

**Background:**

This study aimed to examine changes in obesity rates and obesity-related factors during the COVID-19 pandemic compared to a previous period.

**Methods:**

An ecological time-series study was designed using the Korean National Health and Nutritional Examination Survey (KNHANES) database from 2014 to 2020. The expected values of obesity rate, physical activity rate, and nutrient intake for 2020 were estimated. The differences between the predicted and actual values for 2020 were also examined. In addition, a multiple logistic regression model was used to examine the changes in obesity and physical activity rates in 2020 compared to 2019.

**Results:**

The actual obesity rates in 2020 were higher, and the walking and aerobic physical activity rates were lower than the predicted values for the same year. However, the actual resistance training rates in 2020 were higher and the total energy intake was lower than the predicted values for 2020. In the multiple logistic regression model, the odds ratios for obesity, aerobic physical activity, and walking among men in 2020 were 1.29 (95% CI: 1.08 to 1.55), 0.86 (0.74 to 1.01), and 0.84 (0.73 to 0.97), respectively, compared to those in 2019. However, there were no significant differences between the values for women in 2020 and 2019.

**Conclusions:**

This study suggests that the male obesity rate in Korea has significantly increased during the COVID-19 epidemic, mainly due to a decrease in physical activity.

## Introduction

Coronavirus disease 2019 (COVID-19), an infection caused by SARS-CoV-2, was first identified in Wuhan City in December 2019 and subsequently spread worldwide. The World Health Organization declared a public health emergency of international concern in January 2020 and raised it to the pandemic level in March 2020 (1). COVID-19 has not only led to the loss of many lives but has also had a great impact on our lifestyle. Many countries implemented lockdowns or social distancing measures to prevent the spread of COVID-19 (2).

Moderate- to vigorous-intensity physical activity not only reduces the risk of cardiovascular disease mortality but also reduces cancer mortality and all-cause mortality (3). In addition, physical activity above the recommended level was associated with a reduction in the risk of death due to COVID-19 as well as the severity of COVID-19 (4). However, several studies have reported that the recent emerging COVID-19 pandemic has led to a decrease in physical activity levels, mainly due to lockdown or social distancing (2, 5, 6). In addition, physical inactivity and restricted access to healthy and necessary nutrients due to COVID-19 measures are expected to negatively affect dietary habits and obesity (7). As expected, a study conducted in the UK in 2020 reported that participants experienced negative changes in eating and physical activity behaviors and barriers to weight management during the lockdown during the COVID-19 pandemic compared to their lifestyle before the lockdown (8). In Poland, it has been reported that the frequency of meals, the proportion of people snacking, and the incidence of weight gain significantly increased during the lockdown due to COVID-19 in 2020 compared to those before the lockdown (9).

However, most previous studies were not representative sampling studies for the entire country or population. In addition, changes in physical activity, nutrient intake, and body weight during lockdowns or the COVID-19 pandemic did not accurately reflect real nationwide changes because they used mobile devices or self-questionnaires. In addition, there were controversial study findings regarding whether physical activity or nutrient intake really had deteriorated during the COVID-19 pandemic (10, 11).

South Korea is geographically close to China, with many personal contacts, including travel and trade. Therefore, the early COVID-19 outbreak could not be avoided. More than 6,600 patients diagnosed with COVID-19 were reported in Dae-Gu City and the Gyeongsangbuk-do region around March 2020, in the early stage of the COVID-19 pandemic (12). However, unlike other countries, South Korea did not implement a lockdown and only maintained non-pharmaceutical interventions (NPI) such as social distancing, hand washing, and wearing a facial mask (13).

Therefore, although South Korea did not implement a compulsory lockdown, we hypothesized that the COVID-19 epidemic affected physical activity, nutrient intake, and the prevalence of obesity resulting from a decrease in outdoor activity due to social distancing and the fear of COVID-19 in South Korea. To test this hypothesis, we used a nationwide representative health survey database and compared it with physical activity rates, nutrient intake, and obesity rates with past trends.

## Materials and methods

### Data source

We used information from the Korean National Health and Nutritional Examination Survey (KNHANES) database from 2014 to 2020 to determine changes in physical activity and prevalence rates of obesity in South Korea. The KNHANES dataset is a representative cross-sectional survey of the entire Korean population conducted by the Korean Center for Disease Control and Prevention (KCDC) from 1998 to 2020 (14). The purpose of this nationwide survey was to estimate national representative and reliable statistics such as health behaviors, prevalence of chronic diseases, and food and nutritional intake at a national health level. The study participants were sampled using a stratified cluster random sampling method to represent the entire Korean population. The protocol and method for this survey were quality-controlled by external experts and the KCDC, the details of which have been previously reported (14, 15). Our study included only adults aged over 20 years old, and the numbers of participants in 2019 and 2020 were 6,542 and 6,072, respectively. The KNHANES dataset is freely available to all researchers. The observational study protocol was reviewed and approved by the Institutional Review Board of Kyung Hee University (KHSIRB-22-114(RA)).

### Measurement of physical activity

Physical activity was measured using the Global Physical Activity Questionnaire (GPAQ) in KNHANES from 2014 to 2020 (16). In the KNHANES, the GPAQ consisted of physical activity in three different domains: activity at work, activity from transportation, and leisure time activity. We converted the metabolic equivalent (MET) minutes per week for physical activity in each domain. We then added all METs and classified physical activity into the following six subdomains: vigorous work, moderate work, travel, vigorous recreation, moderate recreation, and sitting (17). Finally, people who met the following conditions were classified as engaging in aerobic physical activity during work, transport, and leisure time: moderate-intensity physical activity for 150 min or more per week, high-intensity physical activity for 75 min or more per week, or moderate- to- vigorous-intensity physical activity for at least 600 MET minutes or more (18).

The resistance exercise rate (%) was defined as the proportion of those who performed resistance training, such as push-ups, sit-ups, and dumbbells, more than 2 days per week (18). The walking rate (%) was defined as the proportion of walking for more than 30 min for more than 5 days per week (19). Sedentary time was defined as the average time spent lying down or sitting per day.

### Definition of obesity

Obesity was defined as a body mass index (BMI) ≥25 kg/m^2^ according to the Asian-Pacific cutoff points and the 2018 guidelines from the Korean Society for the Study of Obesity (20). Height and weight were measured while the subjects wore a medical gown. Height (m) was measured using a Seca 225 (Seca, Germany). When measuring height after inhaling, the heel, buttocks, back, and head were all in contact with a vertical plate. Body weight (kg) was measured using a GL-6000-20 (G-Tech, Korea). When measuring the weight, participants took off their shoes, and the weight was read to one decimal point while the participant inhaled.

### Other covariates

Total energy intake (kcal/day) and saturated fat intake (g/day) were calculated through a 24-h recall food intake survey for each individual (21). Income level was calculated as the individual income quartile, and education levels were classified as elementary school education, middle school education, high school education, university education, or higher education.

### Statistical analysis

All analyses were performed using a complex survey design by sex. Age standardization was applied to adjust for different age structures by year, using the mid-2005 Korean population as the standard population. For age standardization, the direct standardization method was used by applying the prevalence rates by age groups in 5-year intervals (0–4/5–9/…/80–84/85+) for each year to the 2005 mid-year Korean standard population.

To obtain the expected value for 2020, joinpoint regression was applied to prevalence rates such as obesity and physical activity rates (22), and linear regression analysis was applied to continuous variables like total energy intake (kcal), sedentary time (h), and BMI (kg/m^2^) to estimate the predicted value for 2020. Subsequently, the age-adjusted differences between the actual values measured in the KNHANES in 2020 and the predicted values estimated by linear regression and joinpoint regression models were calculated. In addition, we compared the age-adjusted differences between the actual values measured in the KNHANES in 2019 and those measured in 2020. Linear regression models were used to test the differences in continuous variables between 2019 and 2020 after adjusting for age groups, and logistic regression models were used to test the differences in categorical variables between 2019 and 2020 after adjusting for age groups.

Finally, multiple logistic regression models for complex survey designs were used to estimate the odds ratio (OR) and 95% confidence intervals (CIs) of the obesity, walking, and aerobic physical activity rates for 2020, compared to 2019 after adjusting for age, income level, and education level. Statistical significance was set at *p <*0.05. All statistical analyses were performed using SAS version 9.4 (SAS Institute Inc., Cary, NC, USA) and Stata version 17.0 (StataCorp, TX, USA).

## Results

### Difference in physical activity, nutrient intake, and obesity between the expected value and the observed value of 2020

**Figure 1:**
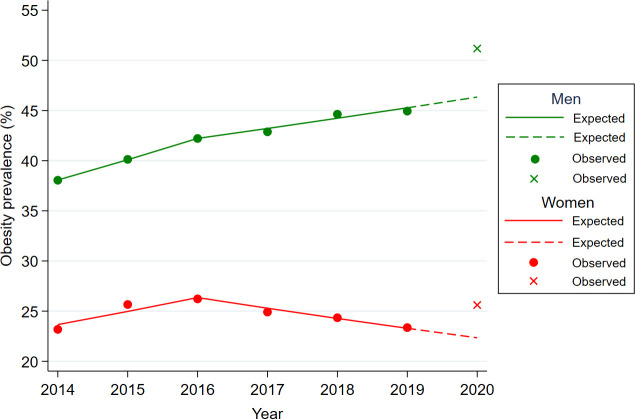
Difference between expected and observed obesity rates for men and women in 2020 year. Footnotes: Age-adjusted obesity rates were used to illustrate for expected and observed obesity rates. The circled dot and the X-point represent the observed obesity rates for each year in KNHANES data from 2014 to 2020 year. The solid represent the expected obesity rates fitted by the Joinpoint regression model using the KNHANES data from 2014 to 2019 year. The dashed line is the predicted value of the obesity rate in 2020 year using the regression equation (y=b1*x + b0) of the Jointpoint regression fitted for the year 2014-2019.

**Figure 2:**
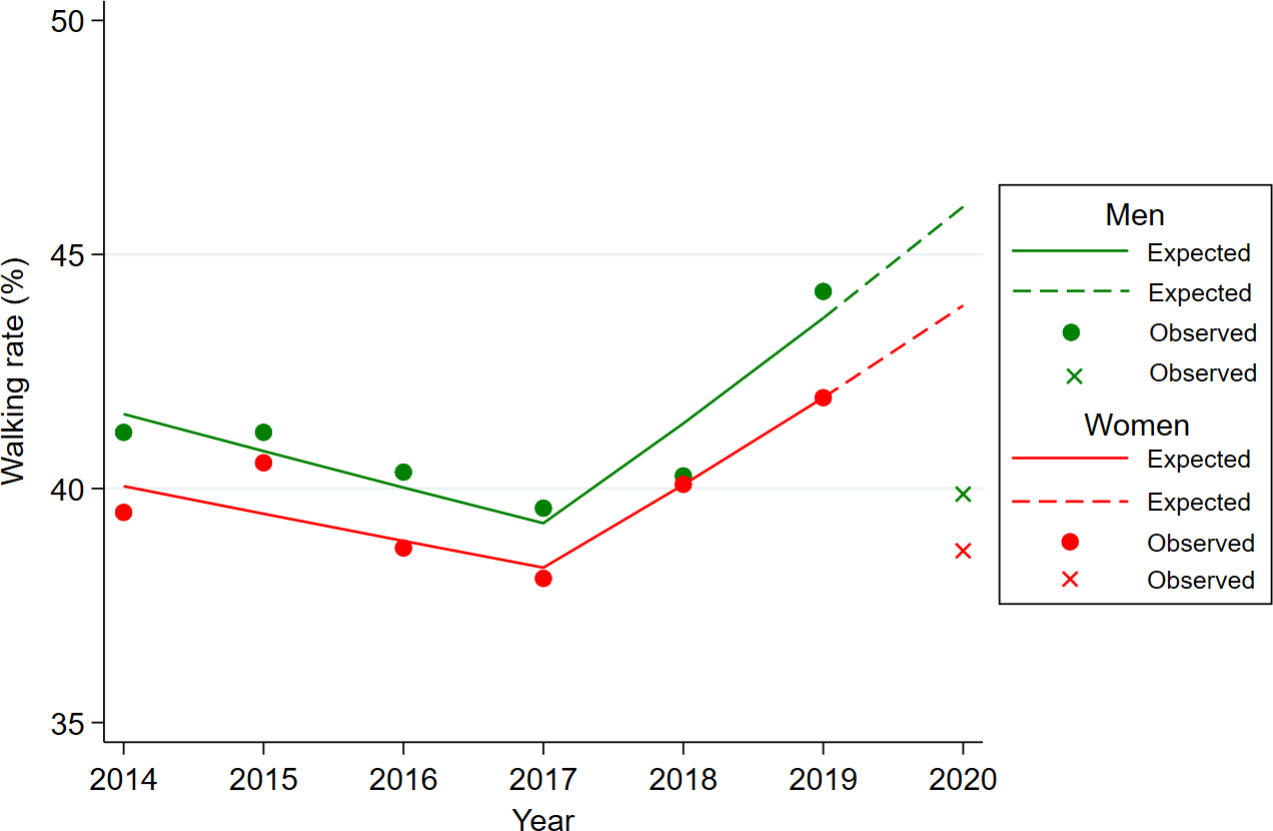
Difference between expected and observed walking rates for men and women in 2020 year. Footnotes: Age-adjusted walking rates were used to illustrate for expected and observed obesity rates. The circled dot and the X-point represent the observed walking rates for each year in KNHANES data from 2014 to 2020 year. The solid represent the expected walking rates fitted by the Joinpoint regression model using the KNHANES data from 2014 to 2019 year. The dashed line is the predicted value of the walking rate in 2020 year using the regression equation (y=b1*x + b0) of the Jointpoint regression fitted for the year 2014-2019.

The expected values for 2020 were predicted using the joinpoint regression model for proportion and the linear regression model for continuous variables, and the expected values for 2020 were compared with the observed values for 2020, which were measured in the KNHANES survey (**Table 1** and **Figures 1**, **2**). The observed obesity rate in 2020 (36.22%) was higher than the expected value (31.82%), and the difference was markedly larger among men (+4.84% vs. +3.27% for women) (**Figure 1**). The observed sedentary time was lower than the expected value.

**Table 1:** Age-adjusted difference between expected and observed values for physical activity, nutrient intake, and obesity in 2020.

Variables	Observed value for 2020	Expected value for 2020	Difference between expected and observed values in 2020
	Age-adjusted mean value or (%) (s.e.)	Age-adjusted mean value or (%)	Age-adjusted mean value or (%)
Total			
Sedentary time (h)	8.68 h (0.08)	8.79 h	−0.11 h
Walking (%)	39.31% (1.02)	45.03%	−5.72%*
Aerobic physical activity (%)	45.33% (0.92)	46.80%	−1.46%
Resistance exercise (%)	24.70% (0.77)	23.59%	+1.11%
Total energy intake (kcal)	1,935.17 kcal (18.84)	1,958.17 kcal	−23.00 kcal
Saturated fat intake (g)	16.79 g (0.03)	17.11 g	−0.31 g*
Obesity (BMI ≥ 25kg/m^2^) (%)	36.22% (0.94)	31.82%	+4.39%*
BMI (kg/m^2^)	24.18 kg/m^2^ (0.08)	23.88 kg/m^2^	0.30 kg/m^2*^
Men			
Sedentary time (h)	8.72 h (0.11)	8.85 h	−0.13 h
Walking (%)	39.88% (1.29)	46.02%	−6.14%*
Aerobic physical activity (%)	47.87% (1.30)	54.09%	−6.22%*
Resistance exercise (%)	31.81% (1.17)	36.33%	+4.53%*
Total energy intake (kcal)	2,264.93 kcal (29.30)	2,309.91 kcal	−44.98 kcal
Saturated fat intake (g)	19.19 g (0.47)	19.76 g	−0.57 g
Obesity (BMI ≥ 25 kg/m^2^) (%)	51.18% (1.64)	46.34%	+4.84%*
BMI (kg/m^2^)	25.18 kg/m^2^ (0.10)	24.68 kg/m^2^	+0.50 kg/m^2^*
Women			
Sedentary time (h)	8.64 h (0.09)	8.73 h	−0.08 h
Walking (%)	38.67% (1.46)	43.91%	−5.24%*
Aerobic physical activity (%)	42.76% (1.20)	41.38%	+1.38%
Resistance exercise (%)	17.34% (0.86)	14.48%	+2.87%*
Total energy intake (kcal)	1,609.64 kcal (19.56)	1,613.89 kcal	−4.25 kcal
Saturated fat intake (g)	14.41 g (0.30)	14.50 g	−0.09 g
Obesity (BMI ≥ 25kg/m^2^) (%)	25.61% (1.11)	22.34%	+3.27%*
BMI (kg/m^2^)	23.08 kg/m^2^ (0.11)	22.99 kg/m^2^	+0.09 kg/m^2^

The observed value for 2020 was actually estimated from the KNHANES database of 2020. Expected value for 2020 year was predicted value using the KNHANES database from 2014 to 2019 fitted by joinpoint regression model and linear regression model (percentage: joinpoint regression model, continuous variables: linear regression model). Differences between expected and observed in 2020 were calculated as follows: (Observed value for 2020 − Expected value for 2020). Walking rate was defined as the proportion of doing walk more than 30 min for more than 5 days per week. Aerobic physical activity was defined as doing moderate-intensity aerobic physical activity for 150 min or more per week or doing vigorous-intensity aerobic physical activity for 75 min or more per week. Resistance exercise rate was defined as the proportion of doing resistance training such as push-ups, sit-ups, and dumbbells, more than 2 days per week.

s.e., standard error.

*The expected value is outside the range of 1.96× standard error of the observed value for 2020.

**Figure 3:**
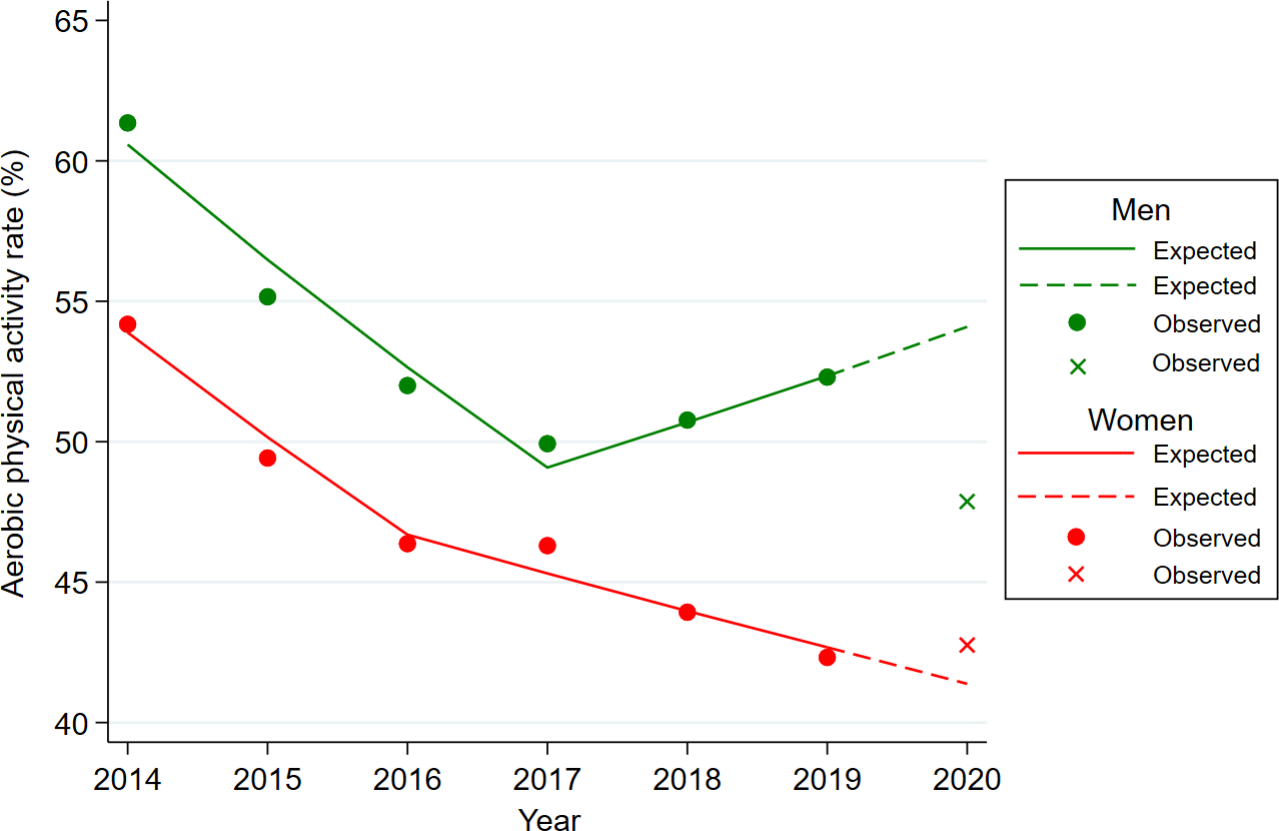
Difference between expected and observed aerobic physical activity rates for men and women in 2020 year. Footnotes: Age-adjusted aerobic physical activity rates were used to illustrate for expected and observed obesity rates. The circled dot and the X-point represent the observed aerobic physical activity rates for each year in KNHANES data from 2014 to 2020 year. The solid represent the expected aerobic physical activity rates fitted by the Joinpoint regression model using the KNHANES data from 2014 to 2019 year. The dashed line is the predicted value of the aerobic physical activity rate in 2020 year using the regression equation (y=b1*x + b0) of the Jointpoint regression fitted for the year 2014-2019.

Regarding physical activity, the observed walking rate was lower than expected for both men and women (**Figure 2**), and the observed aerobic physical activity rate was lower than expected in men but higher than expected in women (**Figure 3**). The observed resistance exercise rate increased more than expected in both men and women. The total energy intake and saturated fat intake were lower than the expected values.

### Change in physical activity, nutrient intake, and obesity in 2020 compared to 2019

In addition to examining the actual change between 2019 and 2020, we compared the observed values in 2019 and 2020 using the KNHANES database (**Table 2**). The actual obesity rates increased in 2020 for men (51.18%) but not for women (25.61%) compared to those in 2019 [difference; men: +6.25% (*p* = 0.005), women: +2.25% (*p* = 0.24)]. The change in actual sedentary time did not show a significant difference between 2019 and 2020.

**Table 2:** Difference in age-standardized rates for physical activity, nutrient intake, and obesity between 2019 and 2020.

Variables	Observed value for 2020	Observed value for 2019	Difference between observed value in 2019 and observed value in 2020	*p*-value*
	Age-adjusted mean value or (%)	Age-adjusted mean value or (%)	Age-adjusted mean value or (%)	
Total				
Sedentary time (h)	8.68 h (0.08)	8.58 h (0.08)	+0.11 h	0.68
Walking (%)	39.31% (1.02)	43.09% (1.02)	−3.78%	0.03*
Aerobic physical activity (%)	45.33% (0.92)	47.49% (0.97)	−2.15%	0.19
Resistance exercise (%)	24.70% (0.77)	23.62% (0.81)	+1.08%	0.35
Total energy intake (kcal)	1,935.17 kcal (18.84)	1,977.67 kcal (19.00)	−42.50 kcal	0.15
Saturated fat intake (g)	16.79 g (0.03)	16.57 g (0.27)	+0.23 g	0.37
Obesity (BMI ≥ 25) (%)	36.22% (0.94)	32.94% (1.01)	+3.28%	0.02*
BMI (kg/m^2^)	24.18 kg/m^2^ (0.08)	23.76 kg/m^2^ (0.07)	+0.42 kg/m^2^	<0.001*
Men				
Sedentary time (h)	8.72 h (0.11)	8.65 h (0.10)	+0.08 h	0.90
Walking (%)	39.88% (1.29)	44.21% (1.36)	−4.33%	0.02*
Aerobic physical activity (%)	47.87% (1.30)	52.30% (1.27)	−4.43%	0.09
Resistance exercise (%)	31.81% (1.17)	32.37% (1.31)	−0.57%	0.72
Total energy intake (kcal)	2,264.93 kcal (29.30)	2,335.72 kcal (27.3)	−70.79 kcal	0.09
Saturated fat intake (g)	19.19 g (0.47)	19.30 g (0.42)	−0.10g	0.98
Obesity (BMI ≥ 25) (%)	51.18% (1.64)	44.94% (1.75)	+6.25%	0.005*
BMI (kg/m^2^)	25.18 kg/m^2^ (0.10)	24.53 kg/m^2^ (0.09)	+0.65 kg/m^2^	<0.001*
Women				
Sedentary time (h)	8.64 h (0.09)	8.51 h (0.09)	+0.14 h	0.79
Walking (%)	38.67% (1.46)	41.94% (1.20)	−3.27%	0.19
Aerobic physical activity (%)	42.76% (1.20)	42.32% (1.25)	+0.44%	0.77
Resistance exercise (%)	17.34% (0.86)	14.59% (0.85)	+2.75%	0.03*
Total energy intake (kcal)	1,609.64 kcal (19.56)	1,630.36 kcal (18.55)	−20.72 kcal	0.62
Saturated fat intake (g)	14.41g (0.30)	13.89 g (0.27)	+0.52 g	0.09
Obesity (BMI ≥ 25) (%)	25.61% (1.11)	23.36% (1.00)	+2.25%	0.24
BMI (kg/m^2^)	23.08 kg/m^2^ (0.11)	22.91 kg/m^2^ (0.10)	+0.17 kg/m^2^	0.30

Walking rate was defined as the proportion of doing walk more than 30 min for more than 5 days per week. Aerobic physical activity was defined as doing moderate-intensity aerobic physical activity for 150 min or more per week or doing vigorous-intensity aerobic physical activity for 75 min or more per week. Resistance exercise rate was defined as the proportion of doing resistance training such as push-ups, sit-ups, and dumbbells, more than 2 days per week.

*The differences in variables between 2019 and 2020 were tested by using linear regression for continuous variables and logistic regression analysis for categorical variables adjusted by age.

The walking rate in 2020 was significantly lower than that in 2019 for men (difference: −4.33%, *p* = 0.002). Although the change in aerobic physical activities was not significant, the aerobic physical activity rate in 2020 was lower than that in 2019 in men and higher than that in 2019 in women. However, the resistance exercise rate for women in 2020 was significantly higher than that in 2019 (difference: +2.75%, *p* = 0.03). There were no statistically significant changes in the total energy intake and saturated fat intake between 2019 and 2020.

### Multiple logistic regression model for obesity and aerobic physical activity in 2020 compared to 2019

After adjusting for age, income level, and education level, a multiple logistic regression analysis was performed by sex to estimate the ORs of obesity rate, aerobic physical activity rate, and walking rate in 2020 compared to 2019 (**Table 3**). For men, compared to 2019, the OR for obesity rates in 2020 was 1.29 times (95% CI: 1.08–1.55) higher even after adjusting for age, income, and education level. In addition, the OR for walking rates among men in 2020 was 0.84 times (95% CI: 0.73–0.97) lower than that in 2019, after adjusting for all covariates. Although it was not statistically significant in men, the OR for aerobic physical activity rate in 2020 was 0.86 (95% CI: 0.74–1.01) compared to that in 2019. However, there were no statistically significant increases or decreases in the ORs for obesity rate, aerobic physical activity rate, and walking rate in 2020 compared to those in 2019 for women.

**Table 3:** Multiple logistic regression model for obesity and aerobic physical activity in 2020 compared to 2019.

	Unadjusted model	Age-adjusted model	Age- and SES-adjusted model
	OR (95% CI)	OR (95% CI)	OR (95% CI)
Men Obesity			
2019	1.00 (reference)	1.00 (reference)	1.00 (reference)
2020	1.27 (1.07–1.50)	1.28 (1.08–1.52)	1.29 (1.08–1.55)
Aerobic physical activity			
2019	1.00 (reference)	1.00 (reference)	1.00 (reference)
2020	0.88 (0.75–1.03)	0.87 (0.74–1.02)	0.86 (0.74–1.01)
Walking			
2019	1.00 (reference)	1.00 (reference)	1.00 (reference)
2020	0.84 (0.73–0.98)	0.84 (0.72–0.97)	0.84 (0.73–0.97)
Women Obesity			
2019	1.00 (reference)	1.00 (reference)	1.00 (reference)
2020	1.10 (0.94–1.29)	1.10 (0.94–1.28)	1.08 (0.92–1.26)
Aerobic physical activity			
2019	1.00 (reference)	1.00 (reference)	1.00 (reference)
2020	0.99 (0.86–1.14)	0.98 (0.85–1.13)	0.98 (0.86–1.13)
Walking			
2019	1.00 (reference)	1.00 (reference)	1.00 (reference)
2020	0.91 (0.79–1.06)	0.91 (0.78–1.05)	0.90 (0.78–1.04)

Socioeconomic status (SES) was defined as quartile income level and education level. Income level was calculated as the individual income quartile and education level was classified as elementary school education, middle school education, high school education, and university education, or higher education. Multiple logistic regression models for obesity, aerobic physical activity, and walking in 2020 were compared with obesity, aerobic physical activity, and walking in 2019, after adjusting for age or age, income level, and education level.

## Discussion

This study examined the changes in obesity and obesity-related factors, such as physical activity and nutrient intake, during the COVID-19 epidemic (2020). Our findings showed that the obesity rate during the COVID-19 epidemic had significantly increased compared to the expected obesity rate of 2019, especially in men. This increase in the obesity rate was accompanied by a decrease in physical activity, especially aerobic physical activity and walking rate.

The increase in obesity rates accompanied by a decrease in physical activity during the COVID-19 epidemic was not limited to Korea but has been reported in several countries around the world. The US Behavioral Risk Factor Surveillance System also reported that the obesity rate in March 2020 had increased significantly by 1.1% compared to that in 2019 (23). In addition, it was reported that weight and BMI increased in 32 countries during the first lockdown period compared to those before the lockdown period (24). Obesity rates in Latin America have also increased simultaneously with the COVID-19 epidemic (25). Furthermore, a meta-analysis of eight countries demonstrated that the body weight and BMI of adolescents and children increased during the lockdowns due to COVID-19 (26). Considering our findings and those of previous studies, increases in weight and BMI during the COVID-19 pandemic seem to be another global obesity pandemic (27).

However, previous studies did not provide any clues as to whether a change in nutrient intake caused the rise in obesity or whether a decrease in physical activity was mainly responsible for the obesity pandemic. Interestingly, our study findings showed that physical activity, especially aerobic physical activity and walking, decreased, whereas nutrient intake did not show significant changes considering the trend before the COVID-19 pandemic. However, it is difficult to generalize these results to other countries. While most other countries implemented strong lockdown policies during the early COVID-19 pandemic, South Korea did not enforce lockdowns throughout the entire period of the COVID-19 pandemic (13, 28). Unlike other countries, South Korea did not implement a strong containment policy, including movement restrictions, and social distancing strategies for COVID-19 were based on the voluntary participation of individuals. South Korea has a very advanced food delivery system and access to grocery stores was not restricted. Therefore, accessibility to food in South Korea was not as difficult as it was in other countries during the COVID-19 pandemic. In fact, a nationwide cross-sectional study of Korean adolescents in 2020 reported that the consumption of fast food and carbonated drinks decreased during the pandemic (29). In addition, the proportion of individuals eating breakfast and home-cooked food increased, thus improving unhealthy dietary habits. These results support our finding that saturated fat intake decreased (29). Furthermore, in a cross-sectional study conducted in Italy, a large proportion of participants made efforts to follow dietary recommendations and improve their dietary habits during the lockdown period (10). In contrast, an international online survey reported that the dietary patterns of people became more unhealthy during the lockdown period compared to those before the COVID-19 pandemic (30). These differences in dietary patterns between countries may be partially attributed to differences in dietary habits and culture, the prevalence of COVID-19, degree of lockdown, and accessibility to food by country.

Regarding physical activities, our findings showed that the walking rate significantly decreased, while that of people performing resistance exercise increased. We inferred that the frequency and duration of movement may have decreased due to fear of COVID-19 or policies such as social distancing, while the frequency of performing resistance exercises increased to compensate for the lack of daily activities. Although there is no clear evidence to support this inference, it was reported that motivation or perceived effort decreased compared to those before the lockdown, but most people maintained the practice of performing resistance training in the multinational survey (11). In addition, the Korea Youth Risk Behavior Web-based Survey (KYRBWS) study also reported that although vigorous physical activity decreased in 2020 compared to 2019 in Korean adolescents, the frequency of strength exercise increased in 2020 (29). Another international online survey study also reported substantial decreases in physical activity, including walking, moderate-intensity physical activity, and vigorous-intensity physical activity (30).

In our study, walking and aerobic physical activity rates decreased, but resistance exercise rates increased slightly. Unlike physical activity, sedentary behavior did not show a significant change during 2020 compared to the pre-pandemic period. This was contrary to the results of a previous study that reported an increase in sedentary time compared to the pre-pandemic period across all age groups, although the study examined relatively small samples (***n*** = 1,035) (30). In addition, a systematic review of 26 studies on physical activity also reported that sedentary behaviors increased during the pandemic lockdown period compared with those before the lockdown (2). Further research is needed to determine the cause of the differences between South Korea and other countries.

Although this study used the KNHANES database, which was representative of the South Korean population, it may include regions and periods that were not actually affected by the COVID-19 epidemic. In addition, our study may be statistically unstable because we only evaluated the impact of COVID-19 for the year 2020. Future studies are necessary to evaluate the long-term impact of COVID-19 on obesity, physical activity, and nutrient intake, including the year 2021. Regarding nutrient intake, only the total energy intake and saturated fat intake were evaluated in our study, and it is necessary to evaluate whether there were changes in dietary patterns and the intake of other nutrients. Additionally, South Korea had a unique response to the COVID-19 pandemic. Unlike other countries, South Korea did not implement a lockdown but strengthened social distancing measures step-by-step, relying on individual voluntary participation. Therefore, our findings cannot easily be extrapolated to other countries. Nevertheless, this study has a great advantage because it examined not only the changes in obesity rates but also the changes in physical activity and nutrient intake that can affect obesity rates using the reliable and large-volume KNHANES database at the national level.

In conclusion, the obesity rate significantly increased in 2020 when the COVID-19 pandemic began in South Korea, compared to that before the COVID-19 pandemic. This increase in the obesity rate in 2020 was accompanied by a decrease in physical activity, especially aerobic physical activity and walking rates, rather than a change in nutrient intake, especially in men. However, further research is necessary to determine whether this phenomenon is limited to South Korea or whether other countries show similar patterns because South Korea did not implement a containment policy such as a nationwide lockdown.

## Data availability statement

We used the Korean National Health and Nutrition Examination Survey data from 2014 year to 2020 year, which is freely open to researchers at the Korea Centers for Disease Control and Prevention. Any researcher can obtain data from the following site. (Available from: https://knhanes.kdca.go.kr/knhanes/main.do).

## Ethics statement

The studies involving human participants were reviewed and approved by Kyung Hee University (KHSIRB-22-114(RA)). Written informed consent for participation was not required for this study in accordance with the national legislation and the institutional requirements.

## Author contributions

C-MO designed the research. HJY conducted the research. HJY and C-MO analyzed the data. HJY and SRP wrote the paper. T-YY, SKP, JYJ, J-HL, and J-HR interpreted the study findings and critically revised the study protocol and manuscript. C-MO was primarily responsible for the final content. All authors have read and approved the final manuscript.

## Acknowledgments

We used the Korean National Health and Nutritional Examination Survey (KNHANES) database from 2014 to 2020. We would like to thank Editage for the English language editing.

## Conflict of interest

The authors declare that the research was conducted in the absence of any commercial or financial relationships that could be construed as a potential conflict of interest.

## Publisher’s note

All claims expressed in this article are solely those of the authors and do not necessarily represent those of their affiliated organizations, or those of the publisher, the editors and the reviewers. Any product that may be evaluated in this article, or claim that may be made by its manufacturer, is not guaranteed or endorsed by the publisher.
